# Specific Oil Detection by Canines: Discrimination of Fresh Spill Hydrocarbons from Weathered Background Oil in Coastal Environments †

**DOI:** 10.3390/ani16111688

**Published:** 2026-05-31

**Authors:** Paul Bunker, Ed Owens

**Affiliations:** 1 Chiron K9 LLC, 1610 Patton Rd., Somerset, TX 78069, USA; 2 Owens Coastal Consultants, 755 Winslow Way E, Bainbridge Island, WA 98110, USA; ed@owenscoastal.com

**Keywords:** canine olfaction, detection dogs 3, oil spill response, hydrocarbon discrimination, environmental monitoring

## Abstract

Oil Detection Canines (ODCs) have been proven to detect spilled oil in various environments, including targets and residual oil sequestered along shorelines. A limitation within the capability is that an ODC detects and responds to all types of stranded hydrocarbons, including any from historical spills or naturally occurring chronic oils, such as tarballs washed up from offshore seeps. In locations historically known for chronic tar deposits, the value of a K9-supported Shoreline Cleanup Assessment Technique (SCAT) survey is limited when large numbers of tar deposits are present, because the canine detects and responds to all of them. This thoroughness can slow down SCAT surveys, reduce efficiency by requiring investigation of each response, and fatigue the canines from the number of alerts. A project supported by the Texas General Land Office (TGLO) was designed to investigate the ability of a trained ODC to discriminate hydrocarbons on a beach that represent a newly spilled oil while ignoring other potential “background” oils. In 2021, Texas Tech University’s Canine Olfaction Lab conducted a laboratory-based study to investigate canines’ ability to respond to weathered oil samples. This research demonstrated that trained canines could effectively discriminate between weathered oils in a laboratory environment. This knowledge was used to design a field program to conduct monthly surveys with two teams: one involved deploying two canines trained on a sample representative of relatively unweathered spilled oils, handled by citizen-scientist volunteers, and the other involved an ODC trained on a range of oil types. The field survey results show that a Specific Oil Detection Canine (SODC) can discriminate between unweathered oil deposits and other background deposits typical of chronic oiling at the same location. Field surveys indicated that SODCs consistently responded exclusively to target oil samples and disregarded naturally occurring background hydrocarbons, while a generalist ODC responded to all hydrocarbon sources present.

## Simple Summary

Specially trained detection canines can be deployed to support oil spill responses and to detect weathered oil such as tarballs and tar patties. This generalized response can reduce operational efficiency during a spill response in an area containing naturally occurring tarballs and tar patties. This study investigated whether canines can be trained to distinguish between weathered and unweathered oils in a shoreline environment containing naturally occurring tarballs and tar patties. Two canines were trained on unweathered oil samples and evaluated in both laboratory and field settings to determine their ability to distinguish unweathered oil from naturally weathered oil. The two canines consistently demonstrated their ability to detect unweathered oil and ignore all naturally weathered oils. These results demonstrate that canines can be trained to detect only unweathered oils and ignore weathered oils in a shoreline environment. This targeted approach may enable responders to rapidly identify areas affected by new spills, reducing response time, costs, and environmental impact.

## 1. Introduction

Oil Detection Canines (ODCs) have proven capability that has successfully supported research and field deployments [1,2,3,4]. Research has provided robust, extensive data on ODCs’ ability to detect hydrocarbon products. Field deployments have demonstrated that ODCs can perform in real-world settings and have exposed canine teams to situations impossible to simulate in a research facility. A canine’s ability to detect oil on shorelines is one example of this experience, with thousands of confirmed alerts. The Shoreline Cleanup Assessment Technique (SCAT) is a proven methodology for responding to an oil spill incident. With ODCs integrated into the SCAT capability, the term K9-SCAT refers to the ODC enhancement to the SCAT protocols.

In May 2017, an ODC was deployed in Prince William Sound, Alaska, to determine whether a trained canine could detect sequestered oil from the March 1989 Prince William Sound spill. Despite the oil having weathered for 28 years in an exposed shoreline environment, the canine detected subsurface oil at multiple sites [5].

In February 2020, an American Petroleum Institute (API) research trial further demonstrated the ability of ODCs to detect heavily weathered oils in the laboratory and in samples of the same oils placed underground to a depth of 5 m (15 feet) [3].

Marine tar residues result from natural and anthropogenic oil releases, which, when exposed to weathering and other processes, form tarballs, patties, and mats [6]. Marine tar residues wash up along beaches worldwide, including the Texas shoreline, often in large numbers, ranging in length from fractions of a centimeter to several meters [7].

Chiron K9 trains ODCs on the Texas coast, providing an opportunity to locate tarballs in a real-world environment and offering natural and double-blind detection of buried oil. The canines are not trained to locate tarballs before reaching the shoreline. Instead, a generalization process occurs during training, as the canines learn to detect any hydrocarbon sample by being exposed to various oil types. Generalization refers to a canine’s ability to be trained to detect a target odor and respond to similar odors [8]; for an ODC, this involves exposure to and imprinting on a range of oil types rather than a single crude oil or refined product. Generalization is often an advantage, as canines can be trained on stored samples at a training facility and then deployed anywhere in the world to respond to any oil type during a spill response. ODCs have successfully detected various sources of spilled oil in Nova Scotia [9] and Saskatchewan, Canada, and in the USA, including Alaska, Wyoming, California, Florida, and Texas, despite being trained only in Texas.

In locations with chronic oiling, the value of a K9-SCAT survey is limited if the canine detects and responds to all of these “background” oils, as this thoroughness slows SCAT surveys, reduces efficiency by requiring investigation of each response, and fatigues the canines due to the number of alerts. Therefore, an advantage to the traditional K9-SCAT capability would be the ability to differentiate between background or naturally occurring oils on a shoreline and oil released during a specific spill incident.

To evaluate this potential, a research project—Development of Oil-Specific Detection Canine Capability to Differentiate Between Background and Newly Deposited Oils on the Texas Coast—was awarded to the Corpus Christi campus of Texas A&M University (TAMUCC) by the Texas General Land Office (TGLO). This study tests the hypothesis that detection canines trained on specific hydrocarbon profiles can discriminate between recently deposited oil and weathered background oil under operational field conditions.

This new capability is termed the Specific Oil Detection Canine (SODC) to differentiate this application from the traditional Oil Detection Canine. A Specific Oil Detection Canine (SODC) is defined as a detection canine trained to respond to a constrained hydrocarbon odor profile while actively discriminating against non-target hydrocarbons.

In the first phase of this project in 2021, Texas Tech University’s (TTU) Canine Olfaction Laboratory conducted a research trial to investigate the ability of canines to differentiate between several weathered oil samples. This research demonstrated that trained canines could effectively discriminate between different oils within a laboratory environment [10]. The subsequent applied phases of the study evaluated the capability of two SODCs trained in the laboratory to survey a section of Texas shoreline and ignore tarballs and any other existing background oils while responding (alerting) only to oil on which they had been specifically trained (imprinted).

## 2. Materials and Methods

### 2.1. Method: Laboratory Training

Two canines were selected for the SODC study;

- Bin, a male German Shorthaired Pointer. Date of Birth: 24 July 2016.
- Luna, a female German Shorthaired Pointer. Date of Birth: 2 October 2018.

Both were adopted by Chiron K9 from a federal canine training center because they had not met the standards required for federal service. They were trained by Chiron K9 for an explosives detection research project with Texas Tech University’s (TTU) Canine Olfaction Research and Education (CORE) Lab. The training involved teaching the canines how to use an olfactometer(Texas Tech University, TX, USA) [11]. Both canines completed the research study and were then returned to Chiron K9 for reallocation to other projects.

**Figure 1:**
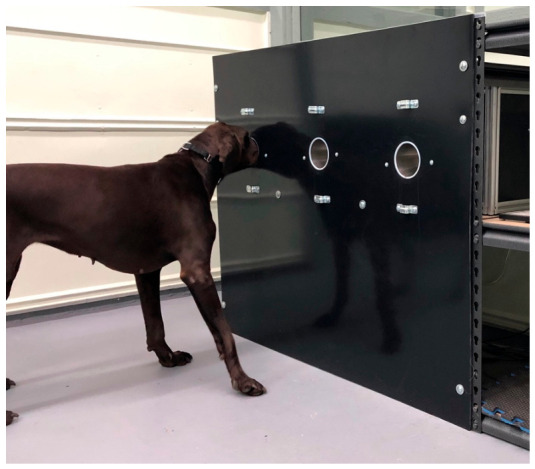
Luna during a Chiron K9 olfactometer test.

Initial training for both canines involved using an olfactometer. Carbon-filtered air from a commercial air pump was controlled by rotameter flow meters and directed via automated solenoid valves to pick up the desired odor in saturation vials. This odor panel consists of three sample ports, each of which receives a different odor by flowing air through an individual vial containing a target, a distracter, or no odor (no target). Only one odor is presented to each sample port at a time. Clean dilution air and odor are mixed in a Teflon (polytetrafluoroethylene [PTFE]) manifold and delivered to the sample port; a computer controls which airstream is delivered to each port and collects canine response data. Once the canine is trained to use the device, the trials are fully automated and considered “double-blind”, with randomness controlled by the computer. An audible cue from the computer signals whether the canine’s response is correct or incorrect. A correct response is when a canine holds its nose within the target port for 4 s. An incorrect response is either a canine holding its nose in a port that does not contain a target odor for 4 s or failing to respond to a port that does contain a target odor. The canine is reinforced with a reward, such as a treat or a toy, for correct responses and not reinforced for incorrect responses [11]. Training to use an olfactometer takes approximately four weeks. Bin had completed oil discrimination research at TTU’s CORE in Phase 1 of this project, so only Luna was trained by Chiron K9 for this step (Figure 1).

Olfactometer training consisted of imprinting (the term used in the canine community), that is, associating a target odor, West Texas Intermediate (WTI) and Bunker C crude oils, with a reinforcement reward. Once this association was established, discrimination of the target from other presented odors was added. Initially, the odors from which the canine was required to discriminate WTI were sand, feathers (collected from a beach), and candy. Weathered oils, including tarballs, were added as the training progressed. After the training was complete, an assessment program was conducted to determine whether Luna could discriminate WTI crude oil from other oils. The assessment consisted of four sets of ten trials conducted in one day. Eighteen vials were prepared, six per olfactometer port. The hydrocarbon samples were mixed into Quikrete Play sand, using the same sand as the unoiled control in one vial (Table 1). This phase took five days to complete.

**Figure 2:**
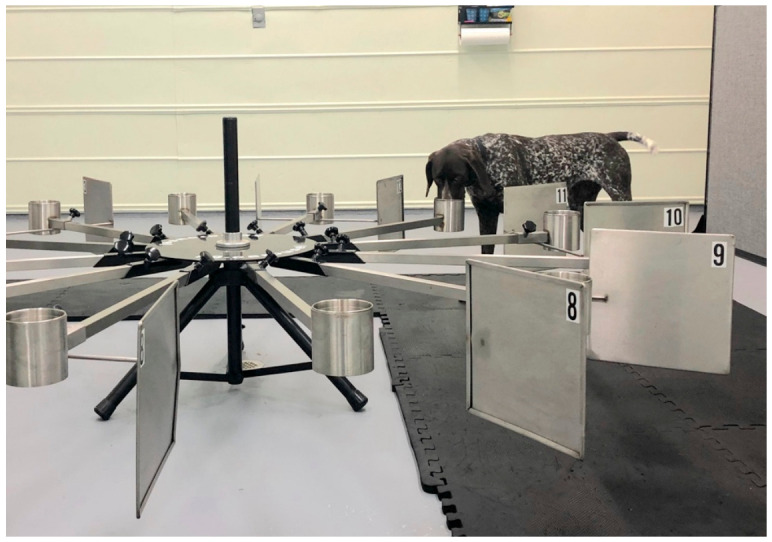
Bin during an odor carousel assessment at the Chiron K9 lab.

After completing the olfactometer phase, both canines, Bin and Luna, continued the same training plan to prepare them for field deployment. This next step involved an odor carousel (Figure 2), a 12-arm stainless-steel wheel with a pot at the end of each arm. Mason jars containing different odor samples were placed inside the pots. For each run, one jar contained a sample of the WTI or Bunker C oils. The remaining eleven jars contained either tarballs or distractor odors, such as beach sand, seawater in the sand, seaweed, and shells, collected from Texas beaches. The canines had to search the carousel pots and respond to the WTI or Bunker C target oil while ignoring the tarballs and distractor odors. At the end of the two-week carousel training phase, the canines were required to successfully complete a progress assessment. The assessment required the canines to complete three double-blind trials searching the carousel. Each trial had one target oil and eleven distractor odors in Mason jars, placed by an assistant trainer. The canine handler was not present in the lab while the carousel was being set up for the trials. The canines were required to search the carousel and correctly respond to the target oil, with no false-positive responses. The position of the target oil was changed for each trial by the assistant trainer spinning the carousel. Once the canines had successfully completed the assessment, they transitioned to the field phase.

### 2.2. Method: Field Surveys

The field activities were conducted by a professional trainer experienced in canine care and ethical use. These observational trials were not considered outside the normal biological functions of working dog training. However, as part of the study protocol, the project was reviewed and approved by the Institutional Animal Care and Use Committee (IACUC) of TAMUCC [13,14]. The training methods for these SODCs are described by Bunker [15].

**Figure 3:**
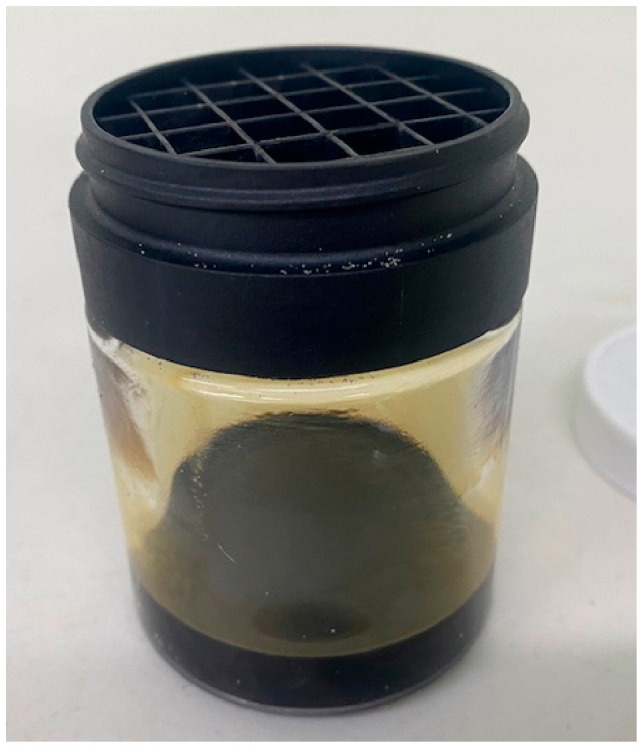
A Training Aid Delivery Device (TADD).

**Figure 4:**
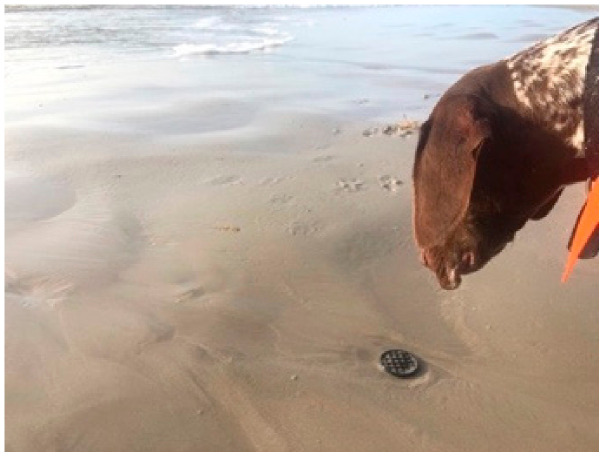
Bin alerts to a TADD containing a target oil placed in the beach sediments.

**Figure 5:**
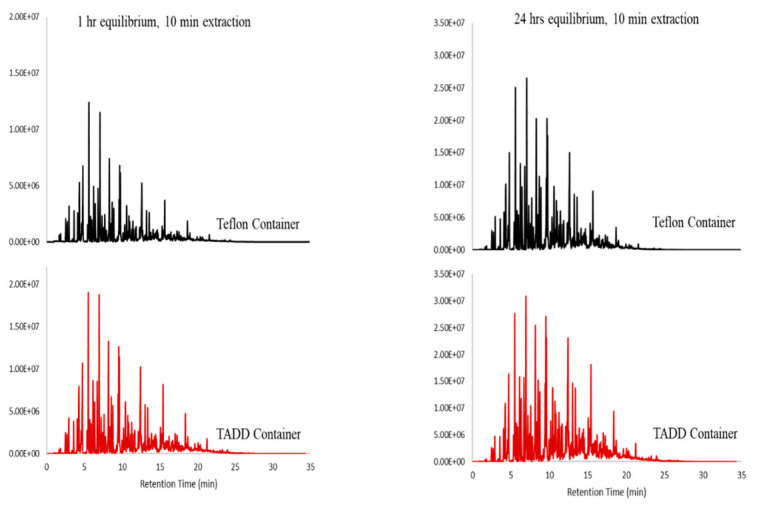
Chromatograms comparing the VOCs collected from a TADD and an open vessel containing WTI crude oil after both 1 h and 24 h equilibration times [17]. Reproduced with permission from Michelle Karpinsky Ph.D., Characterization of the Volatile Organic Compounds Associated with Environmental Contamination to Aid in Detection and Remediation. Ph.D. Dissertation, 2025.

Permits were issued to TAMUCC by Texas Parks and Wildlife for canine field training on beaches within Mustang Island State Park, with the restriction that no training targets (oil) could be placed on the beaches. This restriction was addressed using Training Aid Delivery Devices (TADDs) (Figure 3) to contain the target oils. TADDs were developed by the US Army specifically to safely contain training materials while allowing volatile molecules to escape and be detected by a canine. The TADD is a glass or plastic jar with a lid that contains a hydrophobic and oleophobic chemical-resistant membrane [16]. The membrane allows odor to escape but prevents the contents from coming into contact with the environment. This enabled WTI crude and Bunker C training targets to be deployed on the shoreline without releasing oil into the environment (Figure 4). The TADD membrane does not affect the headspace profile of unweathered oil in chemical analysis (Figure 5)

All training aids (TADDs) were stored, transported, and handled in accordance with industry-standard protocols to prevent cross-contamination. Disposable gloves were used at all times when handling the TADDs. The TADDs were stored in mylar bags and separated from one another within the transportation containers. The holes in the sand into which the TADDs were placed were dug with a stainless-steel trowel, then cleaned and decontaminated after each use.

### 2.3. Field Evaluation Phase

The SODCs were introduced to the TADDs containing target oil samples (Bunker C and WTI), utilizing the odor carousel as in the previous training sessions. This time, TADDs with target oils were used, as well as tarballs inside TADDs and blank (empty) TADDs. The canines were required to search the carousel pots containing TADDs and respond to the WTI or Bunker C target oil while ignoring the tarballs in TADDs and blank TADDs. Once each canine had completed three successful trials with zero false positives and responded to the target odor, the training transitioned to TADDs in the field phase.

The last training step before the field evaluation surveys involved training the canines for an off-leash survey technique termed Wide Area Search (WAS). The system requires the canines to follow a zigzag search pattern off-leash and in front of the trainer, under verbal, hand-signal, and whistle-based directional control. Before the shoreline trials, the canines were required to follow the WAS system under the trainer’s control within the 6-acre outside training area at the Chiron K9 facility. Once the canines could complete a WAS pattern under the trainer’s control, target odors contained in TADDs were placed within the training areas. Each canine completed three separate trials under the trainer’s control and indicated on-target oils within the search area. A TADD with target oil was placed in the area by an assistant trainer without the canine’s handler present. The handler then searched the area with the canine, using the WAS system. The canine was required to detect the TADD-containing target oil and to give no false-negative responses, demonstrating readiness to transition to the shoreline phase.

**Figure 6:**
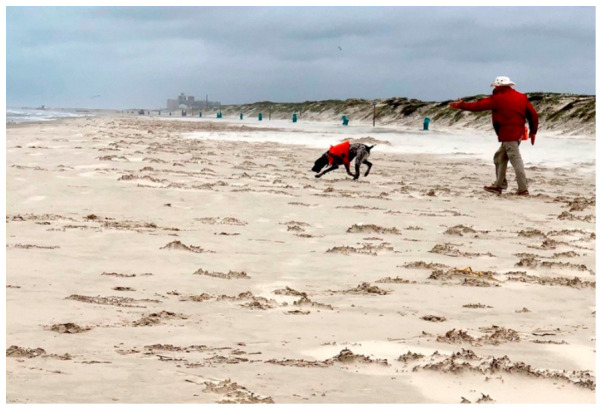
Bin and handler survey a beach using the Wide Area Search technique.

Upon completion of the training phases at the Chiron K9 facility, Bin and Luna were paired with citizen-scientist volunteers from the Corpus Christi area. The volunteers’ field training was conducted on Gulf Coast beaches on Mustang Island, near Corpus Christi, which are known to routinely contain naturally occurring tarballs (Figure 6). The handlers completed an initial two-week training period to ensure they were proficient in handling the canines on the shoreline and conducting WAS surveys.

The canines are worked off leash, and other than providing directional guidance during the WAS, the handlers have no influence on the canines’ reliability in detecting TADDs with target oil. All canines are taught to be “obedient to odor,” a canine community term meaning that if the canine detects its target odor, it is to ignore its handler’s directions unless it receives the emergency recall cue or is reinforced by the handler with a toy for completing the task successfully.

### 2.4. Experimental Design and Data Recording

The study design for the volunteer handlers required searching a predefined shoreline transect area of approximately 6400 m^2^ along a 160 m length of sand shoreline on one day each month. The exact area slightly fluctuates depending on the height of the tide. Each monthly survey consisted of five controlled targets:

- One TADD containing unweathered WTI oil,
- One TADD containing unweathered Bunker C,
- Two TADDs containing Mustang Island tarballs,
- One empty TADD (control).

They were placed randomly by a trainer, without the handlers present, within the defined transect, and evaluated under single-blind conditions. The TADDs were buried just below the sand surface so they could not be seen by the handler. This design provided a consistent ratio of target, distractor, and blank samples across all trials.

The survey program began in May 2022 and was completed in February 2023. For each monthly field survey, TADD training samples were placed on the beach, and the test evaluated whether the SODC would ignore all naturally occurring “background” oils and respond only to the TADDs containing the imprinted oil samples.

Each canine conducted one search of the transect area containing unweathered oils and ignored the other TADDs and all naturally occurring weathered oils.

**Figure 7:**
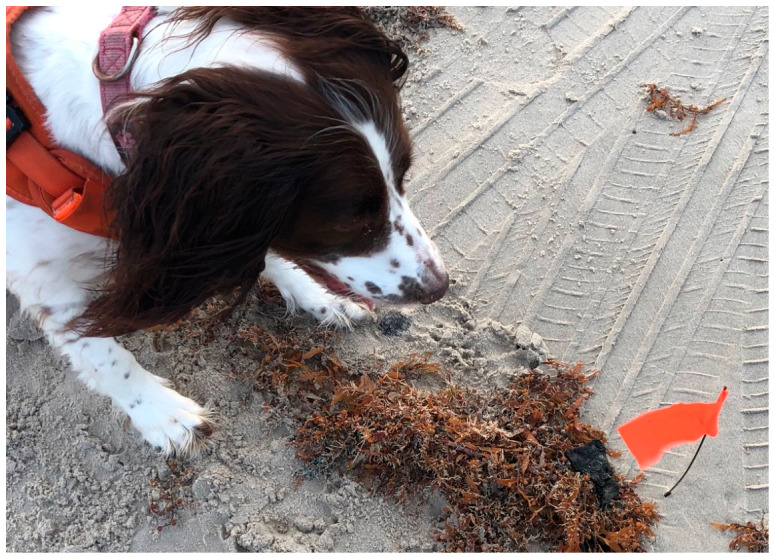
Poppy alerting to a tarball within seaweed.

For this phase of the research, an ODC, Poppy, was used to search the transect after the SODCs had completed their searches. Poppy, being an ODC, is a generalist ODC and detects and alerts to any example of hydrocarbon, weathered or unweathered, within the environment (Figure 7). The same area was searched by the SODC was then searched by the ODC for a functional comparison between trained specificity and generalized detection.

The criteria for success are that the SODCs detect and alert on TADDs containing unweathered oils and ignore other TADDs and naturally occurring weathered oils.

The ODC’s criteria for success were to detect and alert to the TADDs containing weathered and unweathered oils and all naturally occurring weathered oil, plus ignore the empty TADD.

The SODC and ODC did not demonstrate any change in behavior or response to the TADDs that did not contain target oils. Following the SODC survey, the author (PB) conducted a control search using an ODC (Poppy) in the same area.

The TADDs provide a simulated odor plume as a sample of oil on the surface or buried just below the surface (1 cm). As the surface area of the top of the TADD is small (5.8 cm: 2.25 inches), it can only replicate a small oil deposit in the environment. Due to the volatile nature of unweathered oil, in this study, the canines were able to detect the targets at distances greater than 9 m (18 ft) from the source when unburied and follow the plume to the TADD, and 3 m (9 ft) from a buried TADD.

## 3. Results

Monthly beach surveys were conducted by an ODC and volunteer SODC teams along a section of Mustang Island, TX. The surveys with an ODC and SODCs were repeated eight times.

Table 2 summarizes the results for the ODC surveys on days when the SODC teams and the ODC team were deployed.

**Figure 8:**
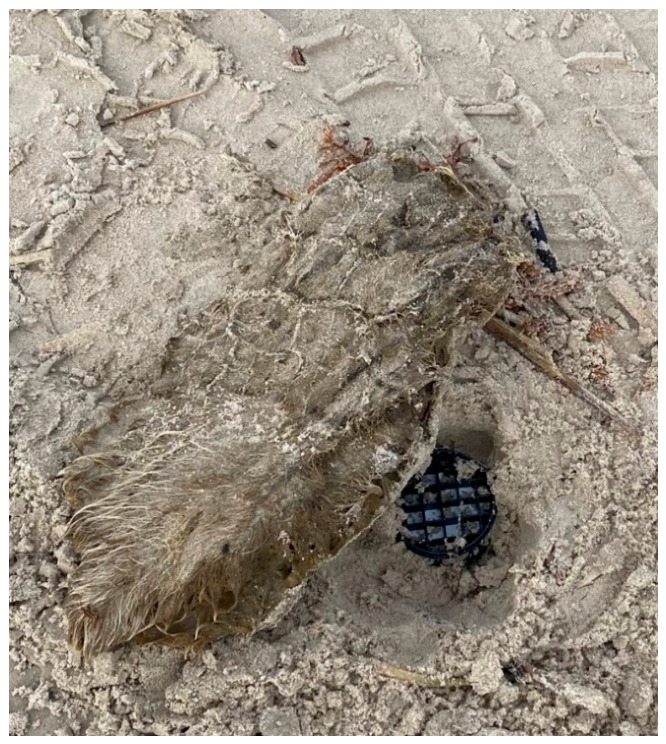
TADD containing WTI crude (28 February 2023).

**Figure 9:**
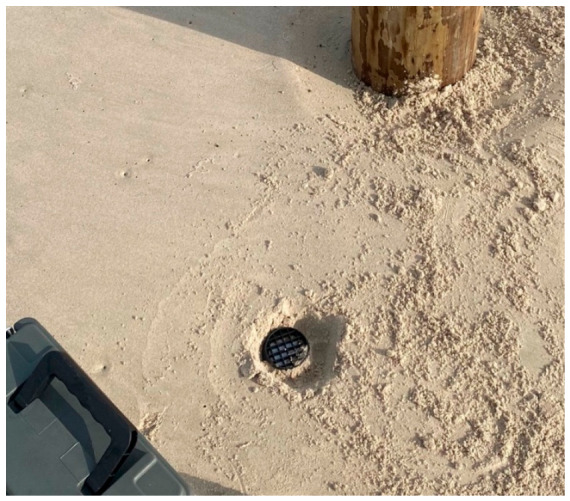
TADD containing Bunker C (28 February 2023).

As an example of the results, on 28 February 2023, a routine survey was conducted by one SODC, Bin, and one ODC, Poppy. Before the SODC team arrived, two TADDs containing imprinted oil samples, one of WTI and one of Bunker C, were buried within the survey area (Figure 8 and Figure 9). 

**Figure 10:**
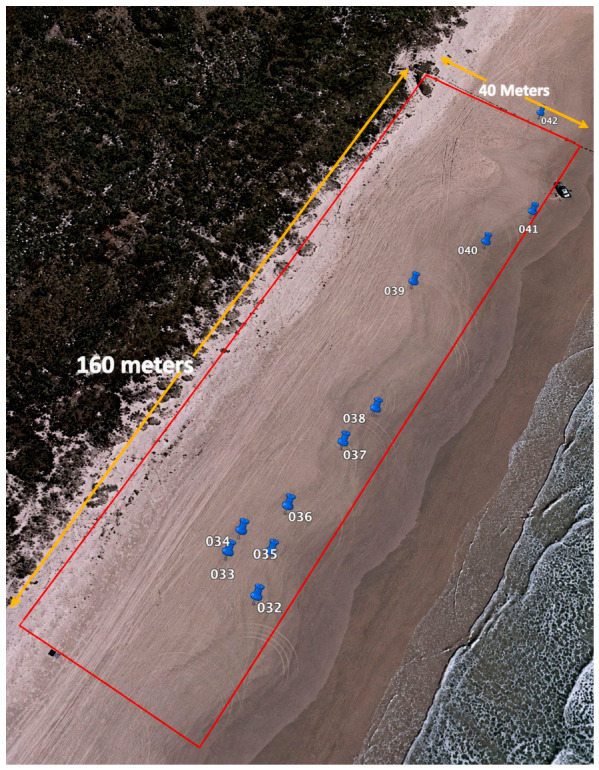
Waypoints of alerts by the ODC Poppy on 28 February 2023.

**Figure 11:**
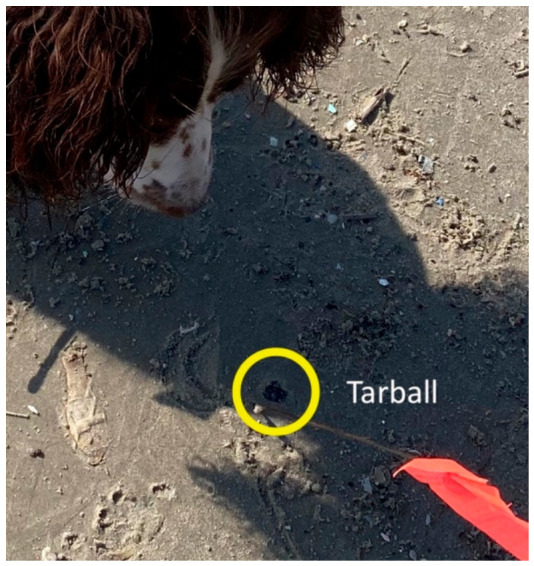
ODC Poppy alerts on a tarball during survey assessment (28 February 2023).

**Figure 12:**
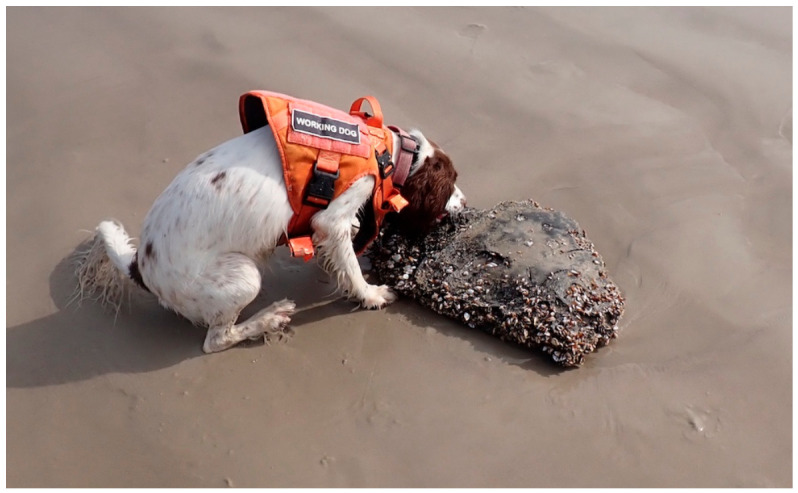
ODC Poppy alerts on a tar pattie being washed ashore (28 February 2023).

Surveys of the transect were conducted by the SODC and the ODC. Waypoints were taken for all alerts by the ODC and SODC (Figure 10). The SODC conducted the first survey and only responded to the two TADDs. After Bin completed a survey, Poppy, the generalist ODC, repeated the survey and responded to eight naturally occurring tarballs (Figure 11), one tar pattie (Figure 12), and two TADDs (Table 3). Bin completed the survey in 8 min and 29 s. Poppy completed the survey in 13 min and 28 s. The total area surveyed was 6400 m^2^ (1.58 acres). There were no false-positive responses from either canine during the survey. Bin detected both samples of the unweathered oil. Although Poppy did respond to eight naturally occurring tarballs and one tar pattie, it is unknown whether there were any false negatives. A false negative occurs when tarballs are present, but the canine does not respond. However, no other weathered oil was observed by the handler or the SCAT Assistant except that detected by the canine.

Across all eight surveys, SODCs consistently detected 100% of target TADDs and produced no alerts for background hydrocarbons, whereas the ODC alerted on all hydrocarbon sources present. This corresponds to a 100% sensitivity for target detection by the SODCs under field conditions, with no observed false-positive responses.

## 4. Discussion

### 4.1. False Positives

**Figure 13:**
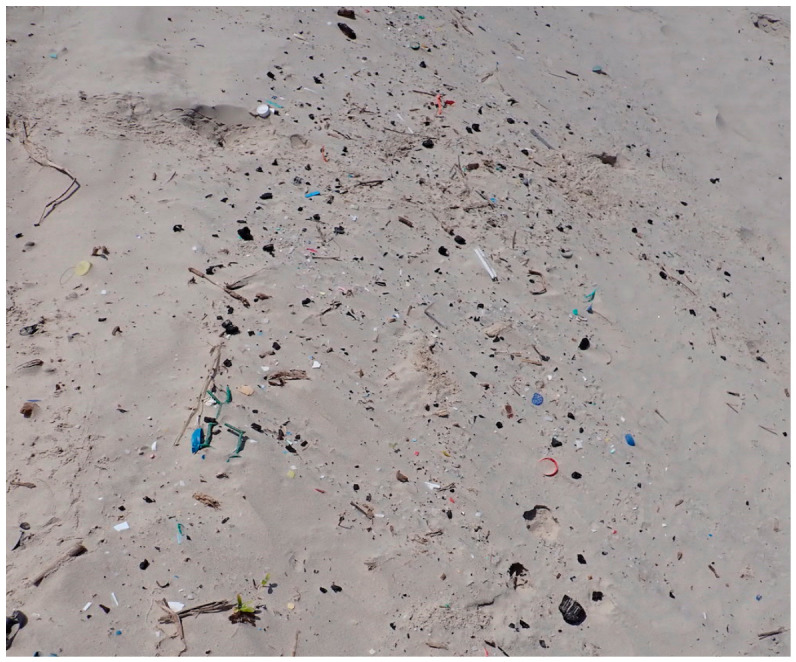
Example of a range of small dark items on the study beach—none were tarballs.

**Figure 14:**
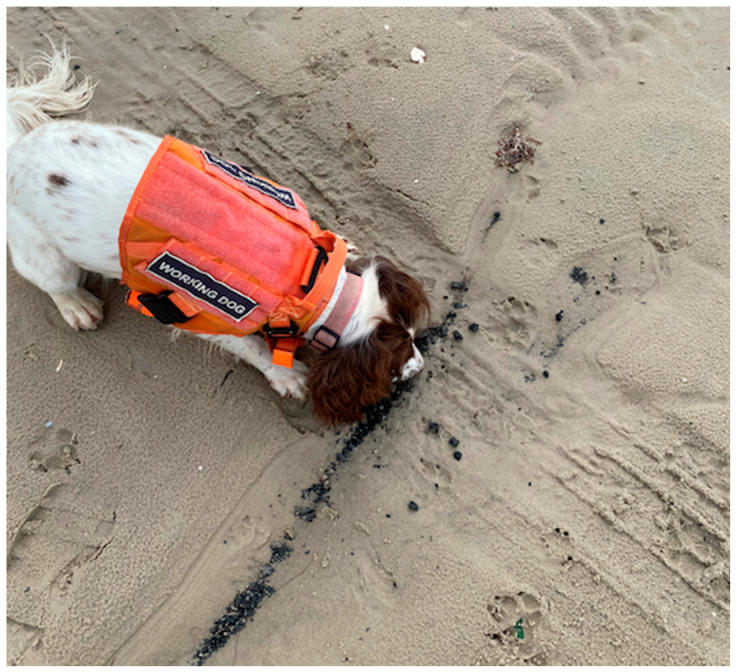
ODC responding to a tarball within a line of charcoal and beach trash.

The ODC and SODC can discriminate oil based on odor profile and ignore visually similar items. An advantage of the ODC and SODC is that the canines can discriminate between target oils and natural and man-made items that resemble weathered oil: “false positives” (Figure 13 and Figure 14). This is an advantage during tarball surveys or for gathering baseline data on an area’s chronic or background oil presence. Dark, non-tar materials, such as small pieces of burnt wood, charcoal, plastic, rubber, or wood, and trash, may resemble tarballs or tar patties in size, shape, and color. During a SCAT survey, these “false positives” can be a significant confounding factor for human observers, as they can be difficult to distinguish even at close range. The human observers would, in most instances, have to pick up and examine individual items to determine whether they are tarballs. The ODC and SODC ignore these false positive materials, which significantly reduces the time required to detect weathered oil on a beach with confusing background items. Because the SODC is trained to ignore tarballs, it would not be used in a tarball survey.

The surveys on Mustang Island found that an average of 22% of all observations of materials by human teams that appeared to be tar or a similar material were, on inspection, not tar (630 false positives out of a total of 2816 observations). In three surveys conducted in the winter of 2024–2025, this number increased to more than half of the observations when the total frequency was <40 items.

### 4.2. Olfaction

**Figure 15:**
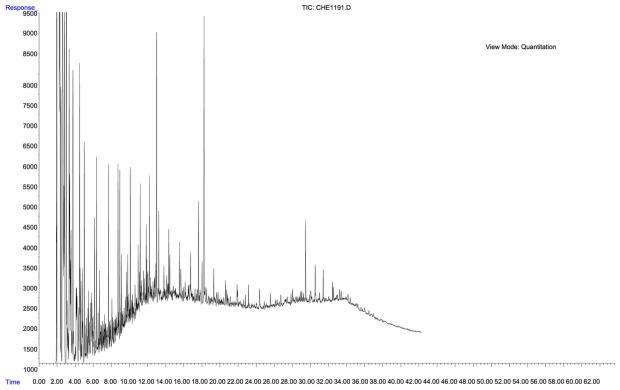
Aliphatic Hydrocarbon/Total Petroleum Hydrocarbon Chromatograms of Bunker C. Reproduced with permission from the American Petroleum Institute [18].

**Figure 16:**
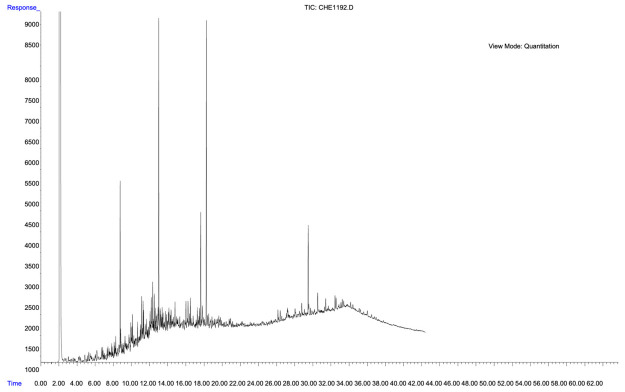
Aliphatic Hydrocarbon/Total Petroleum Hydrocarbon Chromatograms of a Mustang Island tarball Reproduced with permission from the American Petroleum Institute [18].

Unweathered oil is characterized by a relatively strong and complex vapor profile dominated by volatile organic compounds (VOCs), including alkanes and aromatic hydrocarbons such as benzene, toluene, ethylbenzene, and xylenes (BTEX) (Figure 15). These compounds are very volatile and easily evaporate into the air. As evaporation and dissolution remove these volatile compounds, the residual oil becomes depleted of lighter-end molecules. Oxidation and biodegradation continue to transform the chemical matrix, reducing the oil mix’s overall vapor pressure and headspace profile. Therefore, tarballs exhibit a diminished and qualitatively different odor profile, dominated by heavier, less volatile compounds and weathering byproducts (Figure 16). Reduced volatility slows vapor release, resulting in a smaller odor plume than with fresh oil. Additionally, incorporating sand and sediment can physically reduce vapor diffusion, thereby reducing odor availability.

The results of this study indicate that detection canines respond to differences in the volatile profiles of unweathered and weathered oils. However, the precise olfactory mechanism responsible for this discrimination remains unidentified. It remains unclear whether canines respond to the presence of key volatile compounds characteristic of unweathered oil (Figure 15), the absence of compounds lost during weathering (Figure 16), the production of lower odor thresholds by weathered oils, or a combination of these factors. Additionally, it is unclear whether detection is driven by individual compounds or by the overall odor profile of the hydrocarbon mixture. Although the exact mechanism has not been established, both this study and previous laboratory research demonstrate that canines can reliably distinguish between weathered and unweathered oil in both controlled and field environments.

This study demonstrates a transition from generalized detection capability to task-specific olfactory discrimination in operational environments. The results of this study demonstrate SODCs’ ability to distinguish weathered oil from unweathered oil in both laboratory and operational field environments. This capability advances the use of detection canines in the spill response field, and results in a significant enhancement over the traditional ODC in areas where naturally occurring weathered oil is present. This distinction demonstrates that canine detection capability can be tailored to specific operational objectives rather than relying solely on generalized odor detection.

The SODC addresses a gap in response capability, enabling SCAT surveys to be more efficient and reducing the resources and time required to complete a survey. In locations with naturally occurring weathered oils, the ODC may be limited in its functionality due to its generalist capabilities. While the sensitivity of an ODC is needed in most spill response incidents, there are times when the canine’s generalist nature is unnecessary.

The comparison between the SODC and ODC during the field surveys demonstrates the distinction between the specific and generalist nature of the different canines. By understanding these distinctions and deploying the canine teams appropriately, the SCAT response has access to highly sensitive detection technology, but it must be used correctly.

The laboratory findings of this research corroborate the results obtained from field studies. Controlled olfactometer trials have demonstrated that trained dogs can reliably discriminate between hydrocarbon samples without generating false positives [10]. This outcome aligns with previous research from the Canine Olfaction Research and Education Lab at Texas Tech University, which demonstrated that dogs could distinguish unweathered oil samples from weathered ones under controlled conditions. As both studies yielded similar results, the olfactory cues that enable discrimination are robust enough to be detected in laboratory and field environments.

Despite the promising results, it is acknowledged that the sample size of canines was limited. Although the sample size was limited (*n* = 2), consistent replication across multiple trials supports the observed discrimination capability. Field trials were restricted to a single geographic location that featured specific weathered oil types.

It is also recognized that the presentation of unweathered oils is limited to TADD containment systems; therefore, an assessment of canine capabilities in an actual oil spill response would be needed to confirm the study’s findings.

The SODCs demonstrated that they reduce unnecessary alerts from naturally occurring weathered oils, thereby increasing survey efficiency and reducing the time and resources required to investigate every ODC alert. This improves the SCAT decision-making process, increases response speed, and reduces the time required to search areas. A key operational advantage identified in this study is that canines can reduce the frequency of incorrect assessments that result from visual identification of tar-like material.

The high percentage of human false positives (22–50%) compared to zero for the SODC and ODC underscores the advantages of the canine’s discrimination and detection capabilities in support of SCAT.

Future research should investigate the specific point at which a canine determines whether an oil is weathered or unweathered. Additionally, increasing the sample size of canines and evaluating different locations and oil types would enhance understanding of the capability.

This article is a revised and expanded version of a paper entitled “Specific Oil Detection Canines—Shoreline Survey’s Latest Friend” [19], which was presented at the 45th AMOP Technical Seminar in Canada, June 2023.

## 5. Conclusions

The SODC teams are a proven capability that would integrate with K9-SCAT and minimize alerts for naturally occurring hydrocarbons, as they can discriminate between unweathered oil and weathered background deposits. This allows responders to conduct K9-SCAT surveys on shorelines containing chronic oiling residues, such as tarballs, without the canine responding. The capability enhances the SCAT response and provides a fast, efficient, effective, and reliable clearance tool specifically focused on a particular spilled oil.

It is unknown how the canines discriminate between weathered and unweathered oils. One possibility is that canines respond to the absence of volatile compounds lost during weathering or to changes in the overall odor profile they would otherwise expect to find, based on imprint training, such as the lighter BTEXs, or that a heavier compound, such as a PAH, is the primary odor present in the headspace, or the production of lower odor thresholds by weathered oils, or a combination of all these factors

The study was limited to only two SODCs, and although the results were the same for both canines, expanding the study to include more canines would strengthen the data. The research conducted by Texas Tech University’s Canine Olfaction Lab demonstrated, in a laboratory environment, that the canines can reliably discriminate between weathered and unweathered oil samples [10]. This same discrimination capability was replicated in the field using the canines in an applied context.

This capability provides a practical and scalable enhancement to oil spill response operations by enabling targeted detection of relevant hydrocarbon sources while reducing unnecessary search effort.

## Figures and Tables

**Table 1:** Contents of olfactometer vials.

Vial Number	Content	Weight	Comments	Source
1	WTI crude	0.5 gm	Mixed into 4.5 g of sand	Texas crude, Midland, Texas
2	Juniper	0.5 gm	Mixed into 4.5 g of sand	Macondo *
3	CTC	0.5 gm	Mixed into 4.5 g of sand	Macondo **
4	Mustang Island tarball	0.5 gm	Mixed into 4.5 g of sand	Collected by the author (PB) from the shoreline
5	Sand	5 gm		Quikrete Company, Atlanta, GA
6	Blank			

* Juniper: A Macondo crude oil collected on 19 July 2010, by the skimmer vessel USCG Juniper as part of the spill response to the Deepwater Horizon incident on 20 April 2010 [12]. ** CTC: A Macondo crude oil collected on 29 July 2010, by barge number CTC02404 from various skimmer vessels during the spill response of the Deepwater Horizon incident on 20 April 2010 [12]. Luna detected all 40 presentations of the WTI target odor and did not respond falsely to any other odors.

**Table 2:** Confirmed Tarballs and Tar Patties Detected by an Oil Detection Canine (ODC) in the transect area used for the trials. The SODC did not alert to any of the naturally occurring tarballs in the transect area.

	2 May 2022	4 May 2022	26 July 2022	30 August 2022	28 February 2023
Total Tarballs (TB: <10 cm)	52	138	297	Over 1000	8
Total Tar Patties (TOP: >10 cm)	0	0	18	61	1

**Table 3:** Description of targets alerted by the ODC Poppy on 28 February 2023.

Waypoint	Item	Size	Comments	ODC Alert	SODC Alert
32	Tarball	1 cm × 1 cm	Very weathered, hard texture	X	
33	Tarball	0.5 cm × 2 cm	Soft, thin, and pliable	X	
34	Tarball	2 cm × 2 cm	Soft, thin, and pliable (Figure 10)	X	
35	Tarball	1 cm × 1 cm	Very weathered and hard texture	X	
36	Tarball	2 cm × 2 cm	A cluster of six similar tarballs in the same location	X	
37	Tarball	3 cm × 2 cm	Soft, thin, and pliable	X	
38	Tarball	4 cm × 3 cm	Soft, thin, and pliable	X	
39	TADD	10 mL	WTI Crude	X	X
40	Tarball	2 cm × 3 cm	Soft, thin, and pliable	X	
41	Tar pattie	90 cm × 60 cm	Weathered and being washed ashore during the survey. Very hard and brittle with a honeycomb appearance. (Figure 11)	X	
42	TADD	10 mL	Bunker C	X	X

## Data Availability

The raw data supporting the conclusions of this article will be made available by the authors on request.

## Abbreviations

The following abbreviations are used in this manuscript:

BTEX	Benzene, Toluene, Ethylbenzene, and Xylene
IACUC	Institutional Animal Care and Use Committee
ODC	Oil Detection Canine
PAH	Polycyclic aromatic hydrocarbons
PTFE	Polytetrafluoroethylene
SCAT	Shoreline Cleanup Assessment Technique
SODC	Specific Oil Detection Canine
TADD	Training Aid Delivery Device
TAMUCC	Texas A&M University—Corpus Christi
TGLO	Texas General Land Office
TTU	Texas Tech University
VOC	Volatile Organic Compound
WAS	Wide Area Search
WTI	West Texas Intermediate
